# The role of gut microbiota in diabetic peripheral neuropathy rats with cognitive dysfunction

**DOI:** 10.3389/fmicb.2023.1156591

**Published:** 2023-04-18

**Authors:** Wei Huang, Ziqiang Lin, Ailing Sun, JieMin Deng, Anne Manyande, Hongbing Xiang, Gao Feng Zhao, Qingxiong Hong

**Affiliations:** ^1^Department of Anesthesiology, The Second Affiliated Hospital of Guangzhou University of Chinese Medicine, Guangzhou, China; ^2^School of Human and Social Sciences, University of West London, London, United Kingdom; ^3^Department of Anesthesiology, Hubei Key Laboratory of Geriatric Anesthesia and Perioperative Brain Health, Wuhan Clinical Research Center for Geriatric Anesthesia, Tongji Hospital, Tongji Medical College, Huazhong University of Science and Technology, Wuhan, China

**Keywords:** diabetic peripheral neuropathy, cognitive function, gut microbiota, 16S rRNA sequencing, rat

## Abstract

**Introduction:**

Owing to advancements in non-invasive magnetic resonance imaging, many studies have repeatedly showed that diabetes affects the central nervous system in the presence of peripheral neuropathy, suggesting a common or interacting pathological mechanism for both complications.

**Methods:**

We aimed to investigate the role of abnormal gut microbiota in rats with diabetic peripheral neuropathy (DPN) combined with cognitive dysfunction. Glucose-compliant rats with nerve conduction deficits were screened as a successful group of DPN rats. The DPN group was then divided into rats with combined cognitive impairment (CD) and rats with normal cognitive function (NCD) based on the results of the Novel object recognition test. Rat feces were then collected for 16S rRNA gene sequencing of the intestinal flora.

**Results and Discussion:**

The results revealed that abnormalities in *Firmicutes*, *Ruminococcaceae*, *Bacteroidia*, and *Actinobacteria-like microorganisms* may induce DPN complicated by cognitive dysfunction.

## Introduction

Cognitive impairments have been linked to poor glycemic control, long-term duration of Diabetes mellitus (DM), and microvascular complications such as diabetic retinopathy and diabetic peripheral neuropathy (DPN) (Groeneveld et al., 2018). Of these, DPN is the only peripheral nervous system (PNS) disease. With advancements in non-invasive magnetic resonance imaging, studies have indicated that the central nervous system (CNS) is also involved in DPN development (Selvarajah et al., 2011; Lee and Tracey, 2013; Wilkinson et al., 2013). There may be shared pathogenic mechanisms between cognitive impairment and DPN, and several risk factors such as HbA1c levels and chronic hyperglycemic states have been identified for both conditions (Zilliox et al., 2016). However, few studies have examined the co-occurrence of diabetic peripheral nerve complications and cognitive dysfunction.

The intestinal microecosystem, is a significant component of human flora and the largest and most complex microecosystem in the human body. The gut microbiota has been shown to influence disease pathogenesis, including neuroimmune and neurodegenerative diseases, obesity, diabetes, liver disease, and cancer, through the microbiota-gut-brain axis (Iannone et al., 2019; Cryan et al., 2020). In addition, many studies have demonstrated a complex interaction between the gut microbiota and the CNS.

Gut microbiota plays an important role in cognitive function through the gut-brain axis (Gareau, 2014; Rogers et al., 2016). Dysbiosis of the gut microbiota contributes to neuroinflammation, leading to cognitive dysfunction in mice with Parkinson’s disease (PD) (Sampson et al., 2016). Amyloid protein deposition in the intestine changes the level of short-chain fatty acids in the intestinal microflora, which exacerbates cognitive impairment in mice with Alzheimer’s disease (AD) (Zhang et al., 2017). The gut microbiota also impacts cognitive function in patients with AD by affecting the release and metabolism of bile acids (MahmoudianDehkordi et al., 2019) and is associated with other neurological diseases, such as depression (Zheng et al., 2016), seizures (Olson et al., 2018), schizophrenia (Zheng et al., 2019), and Huntington disease especially symptomized with chorea (Radulescu et al., 2019). Similarly, the occurrence and development of DPN is related to intestinal flora imbalance (Gao et al., 2018; Salguero et al., 2019); however, there is little information about the relationship between intestinal flora and cognitive impairment in DPN.

Therefore, we screened DPN rats with concomitant cognitive dysfunction for the first time using a new object recognition assay to compare and analyze the differences between their intestinal flora and those of rats with DPN alone.

## Materials and methods

### Animals

SPF Sprague–Dawley rats weighing 200–220 g were purchased from the Laboratory Animal Management Center of Southern Medical University. Animal production license number: SYXK (Guangdong) 2018–0094, animal qualification number: SCXK (Guangdong) 22,016–0041. The experimental animals were reared according to the feeding and management regulations of SPF Experimental Animal Center, Guangdong Academy of Chinese Medicine. Animal use license number: No. 44002100027214. All operations in the experiment followed the relevant regulations of Animal Ethics Committee of Laboratory Animal Center, Guangdong Academy of Chinese Medicine.

### Modeling

#### DPN model

Rats were randomly divided into the normal control group and DPN groups. After 1 week of adaptive feeding with an ordinary diet, rats in the model group were fed a high-fat and high-sugar diet for 8 weeks and then intraperitoneally injected with 0.1% STZ solution (35 mg/kg). They were then banned from consuming food for 60 h, albeit drinking water. Blood from the tail vein was collected after 72 h, and fasting blood glucose was measured using a blood glucose meter and test strips. When the rat’s blood glucose level was ≥16.7 mmol/L and the blood glucose level was stable for 3 consecutive days, the DM model was judged successful and included in the experiment. Two weeks after the STZ injection, the sciatic nerve conduction velocity of the rats in the DM group was measured. The DPN model was successfully constructed if a nerve conduction disorder occurred in rats that reached the target blood glucose level.

#### Novel object recognition test

The NORTs conducted in our study were based on relevant literature (Tarragon et al., 2014; Li et al., 2022; Liu et al., 2022; Sun et al., 2022; Yang et al., 2022; He et al., 2023). Twenty-four hours before testing or training, the animals were placed in the testing room to acclimatize to the testing environment. The test was divided into training and detection phases. Two identical objects, A and B, were placed symmetrically in the experimental box, and the rat was placed in the field with its back facing the two objects. The distance from the rat’s nose to the two objects was the same. The rat was placed in the box for 10 min, and the video recording device was turned on immediately. The experimenter left the test room, then record the number of times the nose or mouth touched the object and the exploration within a range of 2–3 cm from the object (fore paws resting on an object, sniffing an object, and licking an object were recorded as exploration, and posing or climbing on an object did not count as exploration of a new novel object). One hour after the end of the training period, the experiment in the test period was conducted, in which an object was replaced by a novel object(C) with different shapes and colors but the same size. In different experimental stages and between each task of the two rats, the box and objects were wiped with 70% alcohol to avoid disturbance of the free exploration behavior caused by the smell left by the animals. The exploration time of the rats was calculated by two people watching the video at the same time and finally the average was taken for calculation.

The design process of the novel object recognition experiment is illustrated in the figure below.


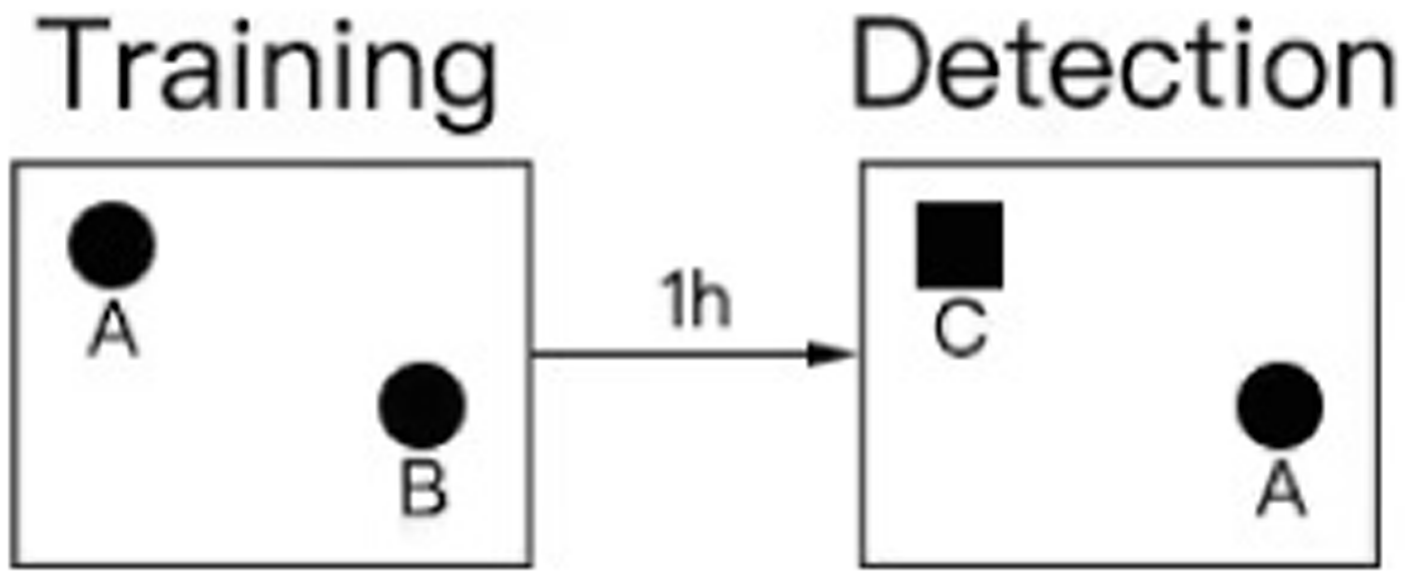


Finally, the recognition index ratio of the time spent exploring object C in the total exploration time was calculated, and SPSS statistical software (IBM, SPSS Inc., NY, United States) was used for cluster analysis to count the cognitive impairment and non-cognitive impairment of novel object recognition in the model group rats. The rats in the modeling group were divided into a CD group with cognitive dysfunction and an NCD group without cognitive dysfunction.

#### Open-field test

Prior to the novel object recognition experiment, an open-field experiment was performed to measure the movement time and distance of the rats, which confirmed that the movement ability of the rats was normal. To eliminate fear, the rats were acclimated to the laboratory for 1 day before the experiment. The rat open-field reaction box was 40 cm high and 100 cm long at the bottom, and the inner walls were painted black. Observation indicators included the number of times the subjects entered the central area, walking distance, time spent in the area, maximum running speed, number of hindlimb stands, and number of times they entered the peripheral area.

#### Animal sample materials

The rats were placed in sterilized cages with Ultraviolet light (UV)-sterilized filter paper, and immediately after natural defecation, feces were collected in sterile Eppendorf (EP) tubes with sterilized tweezers to avoid urine contamination. The collected samples were immediately transferred to a −80°C refrigerator for storage, waiting for analysis. The filter paper was changed every time a rat’s feces were collected to ensure sterility. The collected stool samples were subjected to 16 s rRNA high-throughput sequencing.

### DNA extraction and amplification

Total genomic DNA was extracted using MagPure Soil DNA LQ Kit (Magan) following the manufacturer’s instructions. DNA concentration and integrity were measured with NanoDrop 2000 (Thermo Fisher Scientific, United States) and agarose gel electrophoresis. Extracted DNA was stored at −20°C until further processing. The extracted DNA was used as template for PCR amplification of bacterial 16S rRNA genes with the barcoded primers and Takara Ex Taq (Takara). For bacterial diversity analysis, V3-V4 (or V4-V5) variable regions of 16S rRNA genes was amplified with universal primers 343F (5′-TACGGRAGGCAGCAG-3′) and 798R (5′-AGGGTATCTAATCCT-3′) (Nossa et al., 2010) for V3-V4 regions.

### Library construction and high-throughput sequencing

Libraries were constructed using a library construction kit and quantified using a Qubit instrument and qPCR. Then, after the MiSeq libraries passed quality checks, 16S rRNA gene V3-V4 sequencing was performed on the Illumina MiSeq platform.

### Sequence analysis

The library sequencing and data processing were conducted by OE biotech Co., Ltd. (Shanghai, China). Raw sequencing data were in FASTQ format. Paired-end reads were then preprocessed using Trimmomatic software to detect and cut off ambiguous bases (N). It also cut off low-quality sequences with average quality score below 20 using sliding window trimming approach. After trimming, paired-end reads were assembled using FLASH software. Parameters of assembly were: 10 bp of minimal overlapping, 200 bp of maximum overlapping, and 20% of maximum mismatch rate. Sequences were performed further denoising as follows: reads with ambiguous, homologous sequences or below 200 bp were abandoned. Reads with 75% of bases above Q20 were retained. Then, reads with chimera were detected and removed. These two steps were achieved using QIIME software (version 1.8.0).

Clean reads were subjected to primer sequences removal and clustering to generate operational taxonomic units (OTUs) using Vsearch software with 97% similarity cutoff. The representative read of each OTU was selected using QIIME package. All representative reads were annotated and blasted against Silva database Version 138 using RDP classifier (confidence threshold was 70%).

QIIME software was used for alpha- and beta-diversity analysis. The microbial diversity in samples was estimated using the alpha-diversity that include Chao1 index and Shannon index. The unweighted UniFrac distance matrix performed by R package was used for unweighted UniFrac Principal coordinates analysis (PCoA) to estimate the beta diversity. Then the R package was used to analyze the significant differences between different groups using ANOVA/Kruskal–Wallis statistical test. The linear discriminant analysis effect size (LEfSe) method was used to compare the taxonomy abundance spectrum. LEfSe analysis was calculated using LEfSe software, version V 1.0.8 (Segata et al., 2011).

## Statistical analysis of data

Statistical analysis of all data was performed using SPSS 17 software, and the results were expressed as mean plus or minus standard deviation (MEAN ± SEM). In our analysis of the experimental results, body weight, blood glucose and nerve conduction velocity of control rats and model rats were analyzed by *t*-test under the precondition that the normal distribution was met. The data regarding the new object recognition test in the model group rats were analyzed using hierarchical cluster analysis, dividing the rats in the model group into the CD group and the NCD group. The data regarding the analysis of the locomotor ability of the rats in the CD group and the rats in the NCD group, i.e., the data in the open-field part of the experiment, also conformed to a normal distribution, so the *t*-test was used. *p* < 0.05 was considered to be statistically significant.

## Results

### Changes in animal body weight, blood glucose levels, and nerve conduction velocity

**Figure 1:**
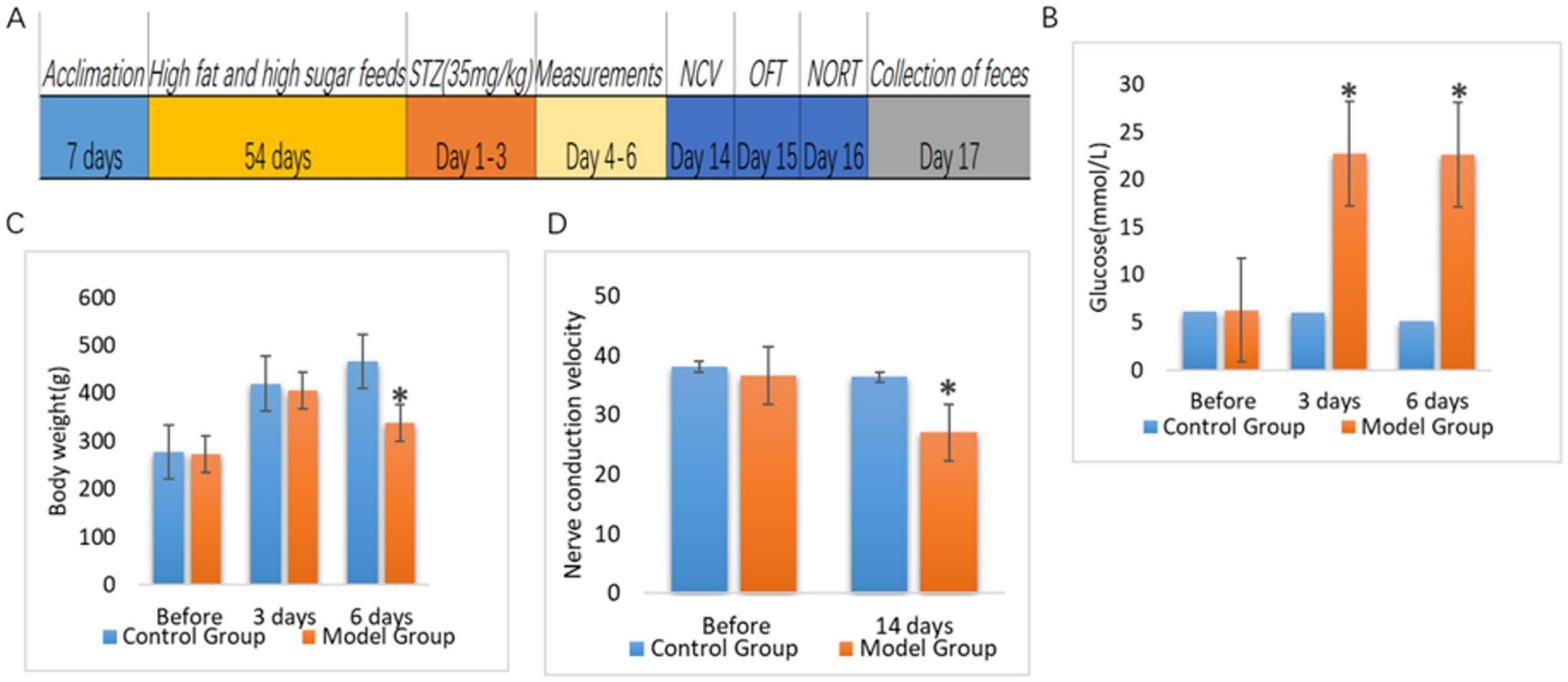
Comparison of blood glucose, body weight and nerve conduction velocity between control rats (Control Group) and rats with diabetic peripheral neuropathy (Model Group). **(A)** Timeline of this study. After 7 days of acclimatization feeding, rats in the experimental group were started on high-fat, high-sugar feeding for 8 weeks. After 8 weeks, rats in the experimental group were injected intraperitoneally with STZ (35 mg/kg) for 3 consecutive days to induce diabetes, and rats in the control group were injected intraperitoneally with saline (35 mg/kg). Body weight and blood glucose levels were measured from day 4 to day 6 after the start of injection, and nerve conduction velocity was measured in the rats on day 14. **(B)** Blood glucose levels (*p* < 0.05). **(C)** Body weight (*p* < 0.05). **(D)** Sciatic nerve conduction velocity (*p* < 0.05). *p<0.05.

After 8 weeks of high-fat and high-sugar feeding, 35 mg/kg streptozotocin (STZ) was injected intraperitoneally for 3 consecutive days to establish type 2 DM (Sharma et al., 2023). We continued the high-fat and high-sugar feeding for 2 weeks; then, the rats were tested for sciatic nerve conduction velocity to establish a DPN model (Figure 1A). There was no significant difference in fasting blood glucose and body weight between control rats (9 rats) and model rats (24 rats) before starting diabetic diet feeding (Figures 1B,C). The blood glucose values of the model rats were significantly higher than those of control rats on days 3 and 6 after the STZ injection (Figure 1B). However, body weight of the rats in the model group was significantly lower than that of the control rats only on day 6 after the STZ injection (Figure 1C). There was no difference in nerve conduction velocity between both groups before the start of the experiment. Two weeks after completion of the intraperitoneal injection of STZ, the sciatic nerve conduction velocity of rats in the model group was significantly lower than that in the control group (Figure 1D).

### Cognitive performance of rats in the DPN group

**Figure 2:**
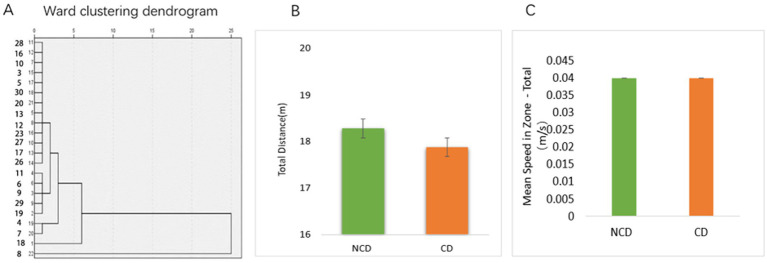
New object recognition in diabetic peripheral neuropathy combined with cognitive impairment (CD) and diabetic peripheral neuropathy non-CD rats. **(A)** Hierarchical cluster analysis of the dendrograms. Results of the hierarchical cluster analysis based on NORT results. DPN rats were divided into cognitive dysfunction group (Group_CD) and no cognitive dysfunction group (Group_NCD). **(B,C)** The NORT test was followed by an absentee field experiment to test the locomotor ability of the rats (*p* > 0.05).

Based on the results of the Novel object recognition test (NORT) behavioral experiment, the model group rats were analyzed by hierarchical clustering into the DPN group with cognitive dysfunction (CD) and DPN group without cognitive dysfunction (NCD) (Figure 2A). After the NORT, an open-field experiment was conducted to compare the total distance and mean locomotor speed of rats in both groups. The results revealed no difference in the locomotor ability of the two groups (Figures 2B,C).

### Analysis of the structure and diversity of the intestinal flora of various groups of rats

**Figure 3:**
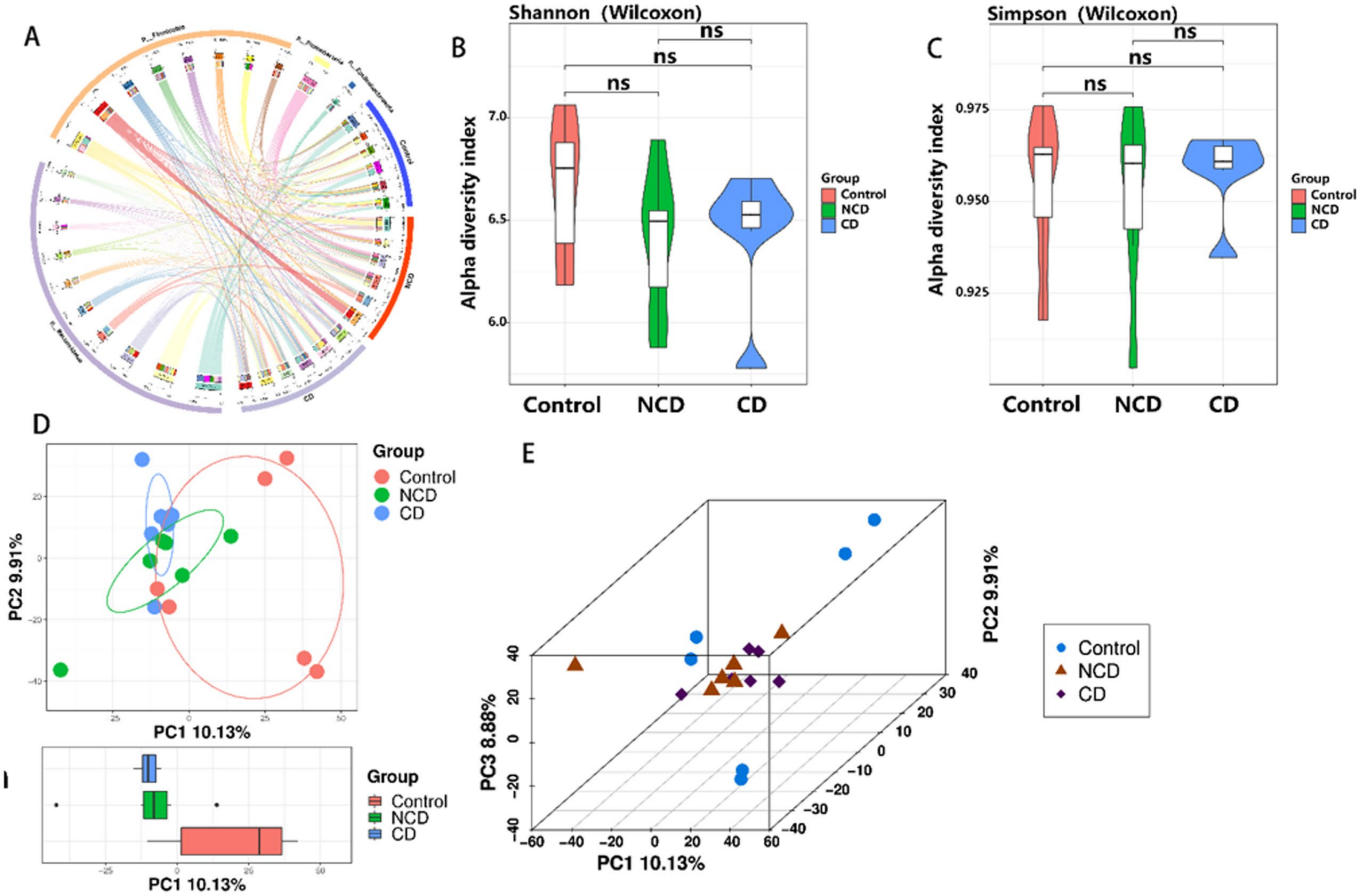
Altered community structure and diversity of the 3 groups of intestinal flora. **(A)** Circos plot shows the different community structures between the three groups. **(B,C)** Alpha-diversity Shannon and Simpson tree indices (*p* > 0.05). **(D)** Boxplot of PCA. **(E)** 3D plot of PCA. Data are presented as mean ± SEM. *p* < 0.05; ns, not significant.

The structure and diversity of the intestinal flora was analyzed comparatively between the CONTROL, CD, and NCD groups by means of Circos plots, alpha-diversity, and beta-diversity. Circos plots showed that the structure of the flora was very different between the three groups, with a significant increase in thick-walled bacteria observed in DPN rats (Figure 3A). For alpha-diversity, the Shannon and Simpson indices indicated no significant variation between the groups (Figures 3B,C). Beta-diversity of PCA showed the gut microbiota to be far apart from each other (Figures 3D,E).

### Changes in intestinal flora richness in DPN rats

Adonis analysis was used to test whether there were significant differences between groups, and the results revealed significant differences in the intestinal flora species (*p* < 0.01) (Supplementary Table 1).

**Figure 4:**
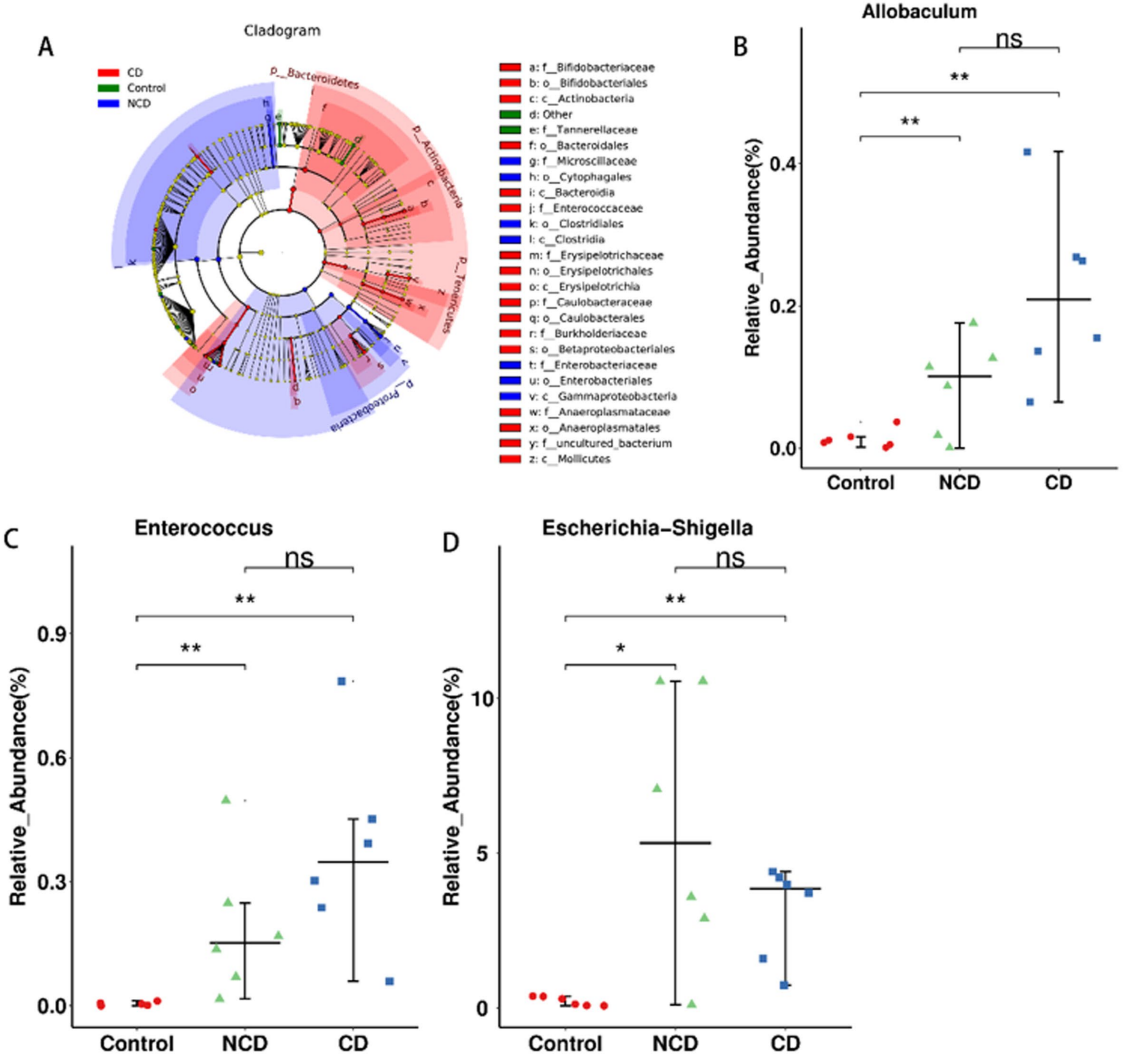
Differentially abundant bacterial taxa between the control and BDL mice. **(A)** LEfSe analysis among the three groups. LefSe, Linear discriminant analysis Effect Size. **(B)** Relative abundance of the phylum Allobaculum. **(C)** Relative abundance of the phylum Enterococcus. **(D)** Relative abundance of the phylum Escherichia-Shigella. Data are presented as the mean ± SEM. *p < 0.05, **p<0.01.

Identification of different gut flora types in the three groups by linear discriminant analysis effect size (LEfSe) analysis (Figure 4A) showed 55 distinguishing characteristics of relative abundance that varied considerably among the three groups. Next, we conducted statistical analyses at each phylum, order, family, genus, and species level. The analysis of fecal samples indicated that 70 bacteria were significantly altered in the three groups (phylum, 5; class, 6; order, 11; family, 14; genus, 23; species, 11). Finally, we selected the relative abundances of the top 10 genera that differed significantly to obtain the abundance of the dominant species within the group for comparison. Compared to the control, DPN rats illustrated significantly increased relative abundance of *Escherichia-Shigella, Ruminococcaceae_UCG-010, Bifidobacterium, Parasutterella, Enterococcus, Anaeroplasma, Allobaculum, Turicibacter, Coprococcus_3,* and *Dubosiella* (Figures 4B–D and Supplementary Figure 1); conversely, there was significantly lower relative abundance of *Ruminiclostridium_6, Parabacteroides, Lachnospiraceae_MK4B4_group, Anaerotruncus,* and *Rikenella* in DPN rats (Supplementary Figure 2). However, the abundance of *Anaeroplasma, Turicibacter,* and *Coprococcus_3* was significantly higher in CD group than in NCD group (Supplementary Figure 3).

### Abundance of differential flora in the CD group of rats

**Figure 5:**
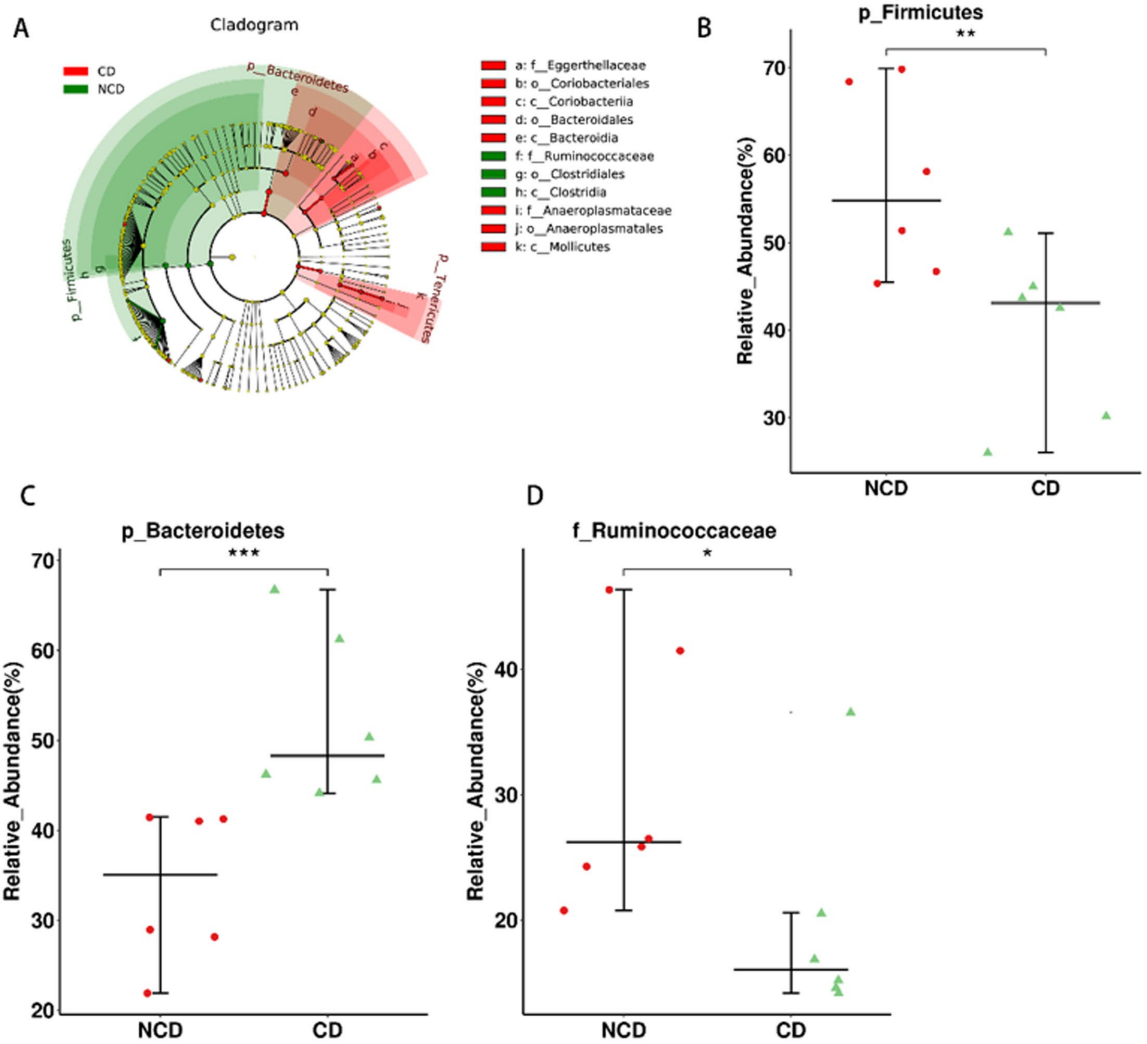
The relationship between dysbiosis markers and cognitive dysfunction. **(A)** Linear discriminant analysis effect size between the Group-CD and Group-NCD groups. **(B–D)** Relative abundance of gut microbiota at the species level. Data are presented as the mean ± SEM. *p < 0.05, **p < 0.01, ***p<0.001.

In the next step of the analysis, we compared the abundance of differential flora in rats in the CD and NCD groups. We performed LEfSe analysis for all *Bacterial* levels between the two groups (Figure 5A). At the species level, we observed that several groups of bacteria in the NCD group, including *c. Clostridia, o. Clostridiales, p. Firmicutes,* and *f. Ruminococcaceae*, were significantly higher based on the linear discriminant analysis (LDA) score [(log10) > 4.30]. Moreover, further analysis using Wilcoxon’s comparative test showed that the differences in the relative abundance of these four groups were statistically significant (*p* < 0.05) (Figures 5B,D and Supplementary Figure 4). In addition to *Anaeroplasma, Turicibacter*, and *Coprococcus_3*, the abundance of these three groups was significantly higher in CD group than in NCD group (Supplementary Figure 3), and *p. Bacteroidetes, c. Bacteroidia, o. Bacteroidales, c. Mollicutes, p. Tenericutes, c. Coriobacteriia,* and *o. Coriobacteriales* were the most abundant microbiota in CD group (LDA score (log10) > 2.18). Three microbiota types, *p. Bacteroidetes, c. Bacteroidia,* and *o. Bacteroidales*, were the most abundant (LDA score (log10) > 4.34) in CD group. Further analysis using Wilcoxon’s comparative test indicate that the differences in the relative abundance of these five groups were statistically significant (*p* < 0.05) (Figure 5C and Supplementary Figure 5). Further comparative analysis of the relative abundance of these nine microbiota types in the three groups using the Kruskal–Wallis algorithm confirmed that only the relative abundance of c*. Clostrid*ia and o. *Clostridiales* was significantly lower in the CD group (Supplementary Figure 6), whereas the relative abundances of *p. Bacteroidetes, c. Bacteroidia, o. Bacteroidales, c. Mollicutes, and p. Tenericutes* were significantly higher in CD group.

## Discussion

The NORT is a valuable measure of cognition mainly used to assess learning and memory based on the differences in exploration time of novel and familiar objects (Lueptow, 2017). We demonstrated that rats in the model group had significantly reduced sciatic nerve conduction velocity 2 weeks after intraperitoneal injection of STZ, along with learning and memory deficits.

The gut microbiota plays an important role in promoting human health. Therefore, exploring the role and mechanisms of gut microbes in DPN combined with cognitive dysfunction is an emerging area of research. Microorganisms colonize the human gut and alter enteric nervous system function by regulating the metabolism and intestinal immune function (Ochoa-Reparaz and Kasper, 2016; Geijselaers et al., 2017; Iliodromiti et al., 2023). Intestinal flora imbalance can lead to the secretion of large amounts of amyloid, lipopolysaccharide (LPS), and inflammation-related molecules and disrupt the intestinal mucosal barrier, which contributes to the activation of microglia and triggers neuroinflammation in the CNS, which may lead to neurodegeneration (Cattaneo et al., 2017; Nguyen et al., 2023).

In this study, there was no significant difference in the alpha-diversity (composed of Shannon and Simpson indices) among the control rats, DPN rats with cognitive impairment, and DPN rats without cognitive impairment.

Previous studies have confirmed that the most predominant microbial taxa in this population are *Firmicutes, Bacteroidetes, Aspergillus, Actinomycetes, and Clostridium*. These bacteria are associated with diabetes, obesity (Jayapala and Lim, 2023), and AD (Patterson et al., 2015; Liu et al., 2020; Barta et al., 2023). A significant reduction in the abundance of thick-walled bacteria and actinomycetes at the phylum level was found in AD patients, in addition to an enrichment of allergic bacteria, which were highly correlated with biomarkers of AD pathology, suggesting that alterations in the AD microbiome may be associated with neuropathological changes in AD (Cenit et al., 2017). The proportion of *Firmicutes* was highest in samples from patients with type 2 diabetes alone (50.43%) and type 2 DPN (61.62%). However, studies have also reported that the abundance of thick-walled bacteria was reduced in the microbiome of patients with type 2 diabetes and AD (Cryan et al., 2020; Doumatey et al., 2020). In our study, the abundance of *Firmicutes* was also significantly reduced in rats in CD group. *Firmicutes* produce butyrate, a health-promoting molecule that is thought to increase insulin sensitivity and exert anti-inflammatory activity (Säemann et al., 2000; den Besten et al., 2013; Udayappan et al., 2016).

In our experiments, we determined that *Ruminococcaceae*, a member of Firmicutes, was also less abundant in CD group than in NCD group; both *Ruminococcaceae* and *Clostridium* are gut microbiota that are beneficial for cognitive function; *Ruminococcaceae* produces butyrate, inhibits histone deacetylase, attenuates the secretion of pro-inflammatory cytokines, and promotes cognitive function (Jiang et al., 2021).

In CD group, the relative abundance of Bacteroidetes was significantly higher. The phylum Bacteroidetes comprises a diverse and abundant group of Gram-negative commensal bacteria in the gut (Rajilić-Stojanović and de Vos, 2014). The main outer membrane component of Gram-negative bacteria is LPS, which stimulates a systemic inflammatory response upon transfer from the gut to the systemic circulation and releases pro-inflammatory cytokines. Dysregulation of the intestinal microecology can promote LPS entry into the systemic circulation by increasing intestinal permeability, leading to inflammation and metabolic dysfunction (Dheer et al., 2016). One study concluded that healthy mice injected directly with LPS developed memory deficits (Frühauf-Perez et al., 2018). The inflammatory response is a possible mechanism for the interaction between gut microbiota and the CNS.

Increased proportions of *Bacteroides, Alistipes, Odoribacter,* and *Barnesiella* have been observed in the microbiome of patients with AD (Angoorani et al., 2022). Recent studies have also revealed a reduction in alpha-diversity and an increase in the thick-walled Bacteries/Bacteroides phylum ratio (an important indicator of gut microbiota health) in AD model mice (Qiao et al., 2022).

An increase in harmful microorganisms, such as Turicibacter and Bacteroides, is implicated in the development of ulcerative colitis (Li et al., 2021; Zhang Y. et al., 2021). The abundance of Turicibacter was positively correlated with the levels of pro-inflammatory cytokines such as interleukin (IL)-6, IL-1β, tumor necrosis factor-alpha (TNF-α), and interferon-gamma (Liang et al., 2019; Dong et al., 2022). By examining the effects of gut microbes on chemotherapy-induced inflammatory and behavioral side effects in female mice, it was shown that the relative abundance of *Turicibacter* spp. was associated with circulating inflammation (IL-1β) and behavioral outcomes (lethargy and anxiety-like behavior). In the absence of antibiotics, a relative increase in *Turicibacter* in the distal colon of chemotherapy-treated mice was associated with reduced motility (Grant et al., 2021). In contrast, fecal microbiota transplantation (FMT) protects against intestinal epithelial barrier disruption induced by dextran sodium sulfate by downregulating the relative abundance of *Turicibacter* (Wen et al., 2021). These findings suggest a complex role of *Turicibacter* in chronic inflammatory environment. Recent studies have proposed that *Turicibacter* can induce the production of intestinal-derived 5-hydroxytryptamine (Dunham et al., 2022), an important neurotransmitter in the PNS and CNS. Its abnormalities in the CNS may be associated with anorexia, stress, psychiatric classification, epilepsy, and dementia. Therefore, abnormalities in relation to *Turicibacter* may play a role in DPN-induced cognitive dysfunction.

We also established that the abundance of actinomycetes in the intestinal flora of CD group was relatively increased, mainly from *Coriobacteriia* and *Coriobacteriales*. Most bacteria belong to the class *Coriobacteriia* anaerobic bacteria, which often exist in the intestinal flora and are pathogenic (Hein et al., 1859). *Coriobacteriales* were a minority in normal mice, accounting for approximately 1% of the detected microorganisms. However, the low proportion of bacteria in this gut microbial ecosystem may be extremely important for host metabolism and should not be overlooked in future research.

*Coriobacteriales*, which are strongly associated with energy production or obesity, are abundant in obese people and obese mice and in mice consuming a “Western diet” or high-fat diet (Goodman et al., 2011). In a high-fat diet mouse model, it was revealed that the balance of *Bifidobacteria/Coriobacteriales* has an important effect on lipid metabolism in mice, and *Bifidobacteria* may indirectly affect lipid metabolism by inhibiting the number of *Coriobacteriales* (Martínez et al., 2009). Claus et al. reported the strongest correlation between red stink bugs and liver triglycerides among Actinomycetes. Furthermore, they found two correlations by calculating the correlation of bacteria in operational taxonomic unit (OTUs) of liver metabolic profiles, with the strongest OTUs belonging to the *Coriobacteriales* family, confirming a link between *Coriobacteriales* and liver triglycerides, glycogen, and glucose (Claus et al., 2011).

An analysis of the gut microbiota of mice exposed to the animal model of depression revealed that *Coriobacteriia* is associated with depressive symptoms in depressive mice. After ketamine treatment, 30 bacteria changed significantly, and the receiver operating characteristic curve showed that Actinobacteria and *Coriobacteriia* might be potential biomarkers of ketamine’s antidepressant efficacy (Huang et al., 2019).

Sleep quality is positively correlated with cognitive function, and sleep disturbance can lead to cognitive impairment. According to previous studies on sleep, *Coriobacteriia* is negatively correlated with total sleep time and sleep efficiency (Zhang R. et al., 2021).

In summary, our findings suggest that gut microbial disorders with persistently elevated abundance of the *phyla Mycobacterium, Turicibacter*, and *Actinobacteria* may be a causal factor in DPN combined with cognitive dysfunction. However, a limitation of this study is that we only performed correlation analysis; thus, it is difficult to determine the causal relationship between gut microbes and cognitive impairment, and the specific mechanisms that induce production need further investigation.

## Data availability statement

The datasets presented in this study can be found in online repositories. The names of the repository/repositories and accession number(s) can be found at: NCBI – BioProjectPRJNA937393.

## Ethics statement

The animal study was reviewed and approved by the Animal Ethics Committee of Laboratory Animal Center, Guangdong Academy of Chinese Medicine.

## Author contributions

QH and GZ contributed to conception and design of the study. ZL and WH organized the database. ZL and AS performed the statistical analysis. WH and JD wrote the first draft of the manuscript. HX and AM wrote the sections of the manuscript. All authors contributed to manuscript revision, read, and approved the submitted version.

## Conflict of interest

The authors declare that the research was conducted in the absence of any commercial or financial relationships that could be construed as a potential conflict of interest.

## Publisher’s note

All claims expressed in this article are solely those of the authors and do not necessarily represent those of their affiliated organizations, or those of the publisher, the editors and the reviewers. Any product that may be evaluated in this article, or claim that may be made by its manufacturer, is not guaranteed or endorsed by the publisher.

## Supplementary material

The Supplementary material for this article can be found online at: https://www.frontiersin.org/articles/10.3389/fmicb.2023.1156591/full#supplementary-material
