# Supplementary material for: The role of gut microbiota in diabetic peripheral neuropathy rats with cognitive dysfunction
Source: Front Microbiol. 2023 Apr 18;14:1156591. doi: 10.3389/fmicb.2023.1156591 (PMC10231493; doi:10.3389/fmicb.2023.1156591)
Supplement: Supplementary file 1 [file Data_Sheet_1.docx]

Supplementary Material

The role of gut microbiota in diabetic peripheral neuropathy rats with cognitive dysfunction

First Author*, Wei Huang^1,a^, Ziqiang Lin^1,a^

*** Correspondence:** *Corresponding Authors: E-mail: [hongqingxiong@gzucm.edu.cn,](mailto:hongqingxiong@gzucm.edu.cn,) [zhaogaofeng_97@163.com(GFZ)](mailto:zhaogaofeng_97@163.com(GFZ)); Telephone: 13560111213; Fax number:020-8188 8362.

# Supplementary Figures

**Supplementary Fig. 1**

Relative abundance of gut microbiota. Data are presented as the mean ± SEM. *p < 0.05; **p < 0.01; ns, not significant.


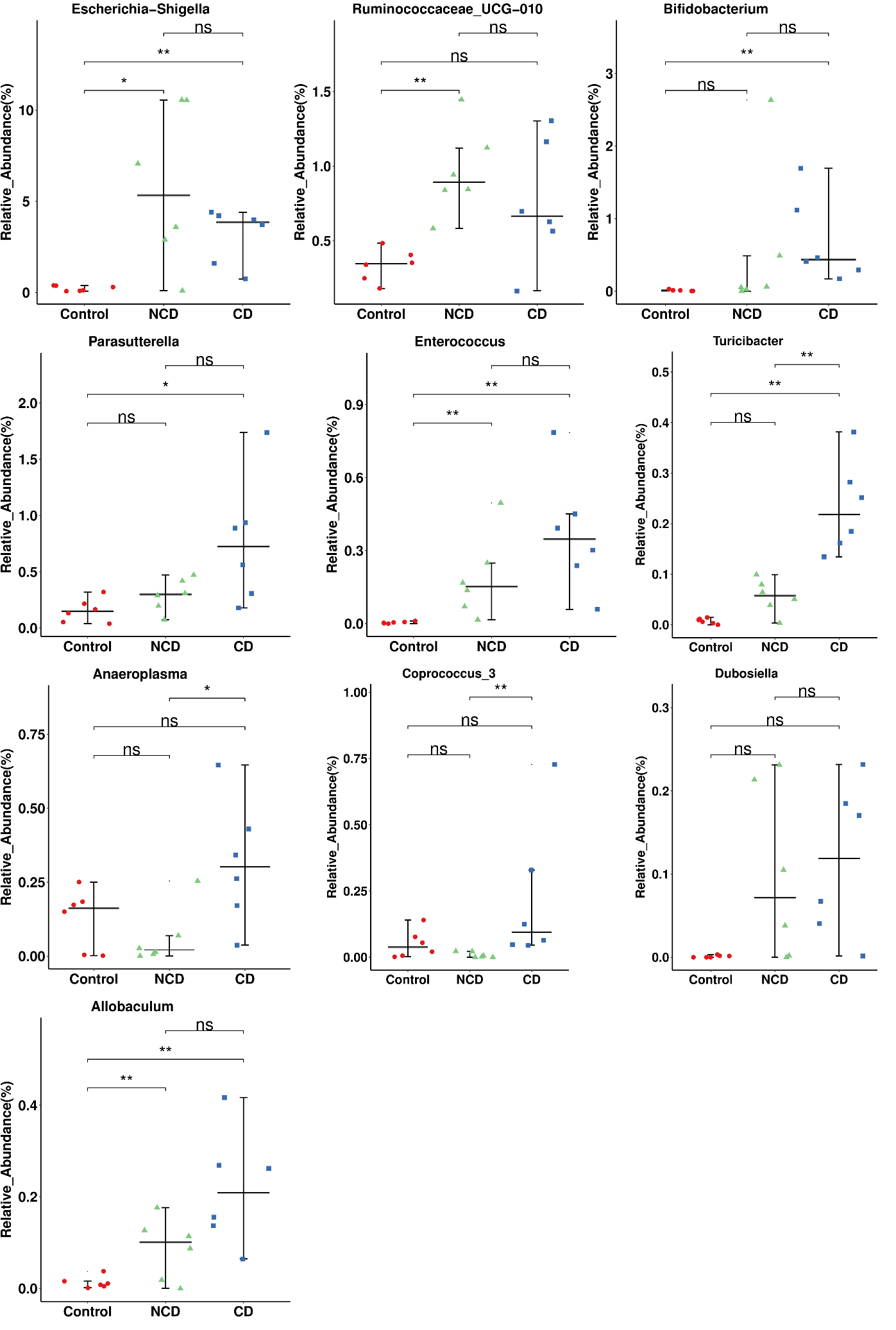


**Supplementary Fig. 2**

Relative abundance of gut microbiota. Data are presented as the mean ± SEM. *p < 0.05; **p < 0.01; ns, not significant.


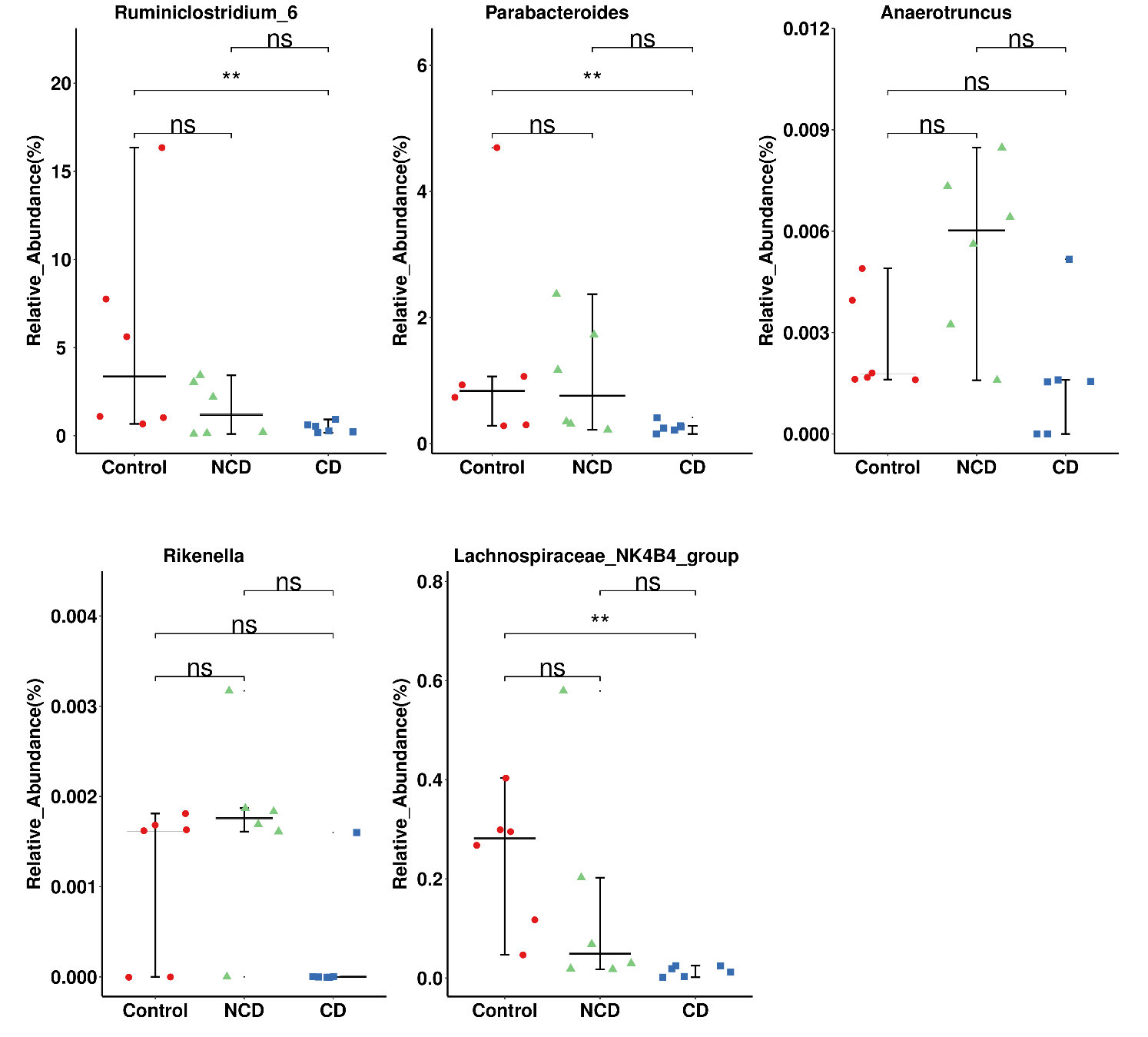


**Supplementary Fig. 3**

Relative abundance of gut microbiota. Data are presented as the mean ± SEM. *p < 0.05; **p < 0.01; ns, not significant.


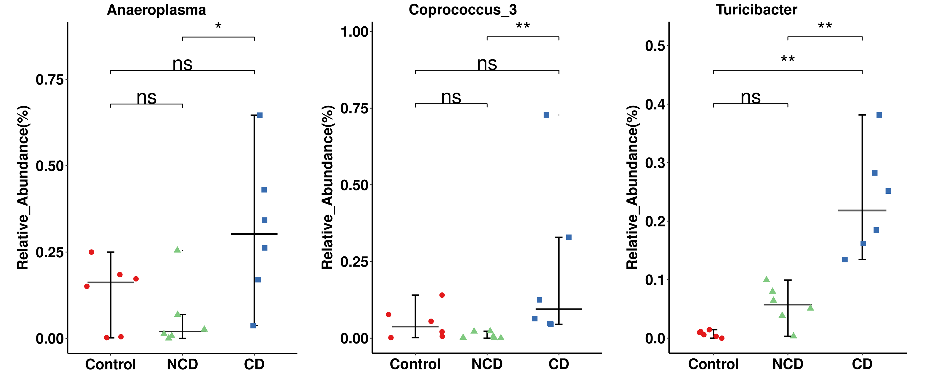


**Supplementary Fig. 4**

Relative abundance of gut microbiota. Data are presented as the mean ± SEM. *p < 0.05; **p < 0.01; ns, not significant.


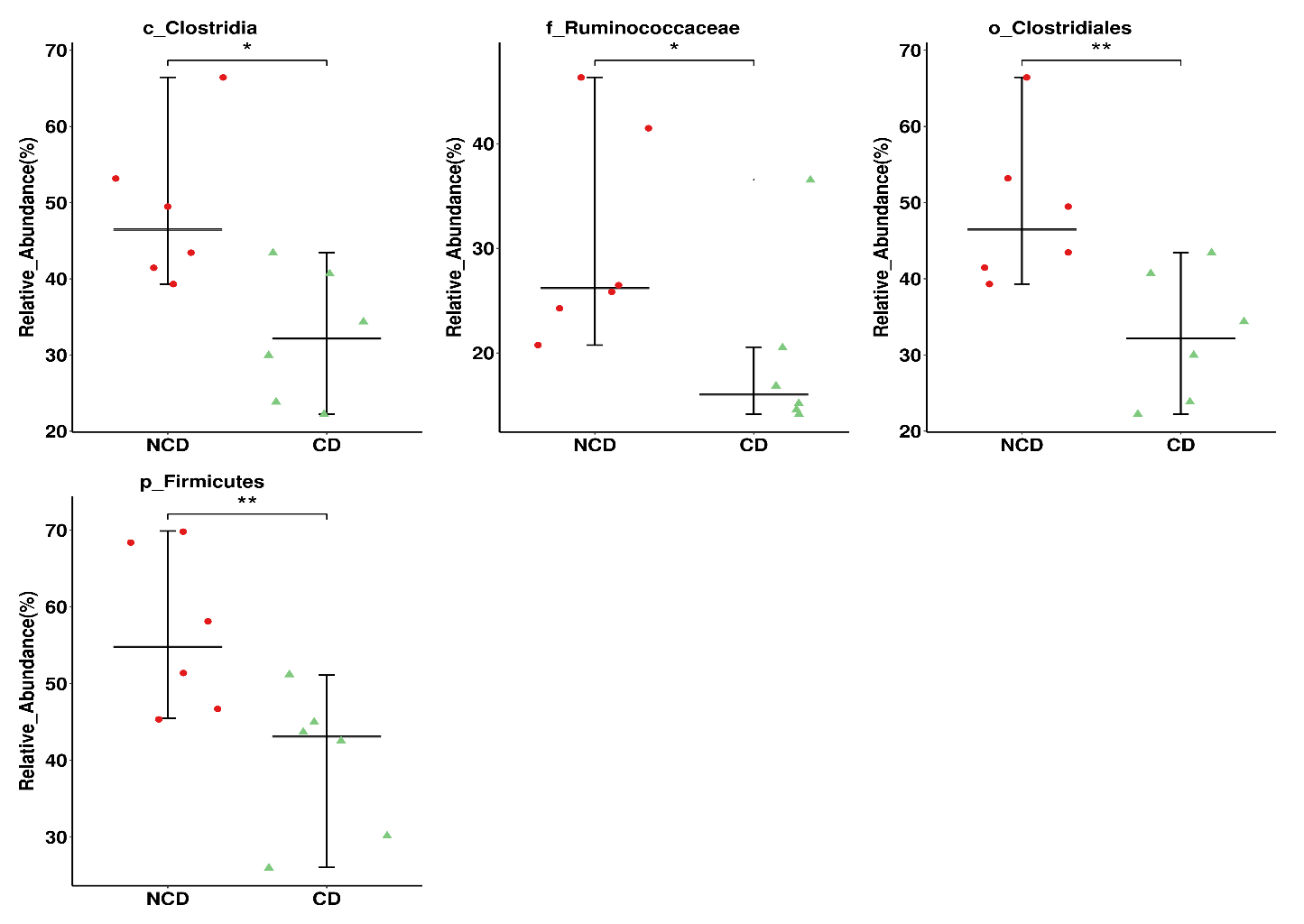


**Supplementary Fig. 5**

Relative abundance of gut microbiota. Data are presented as the mean ± SEM. *p < 0.05; **p < 0.01; ns, not significant.


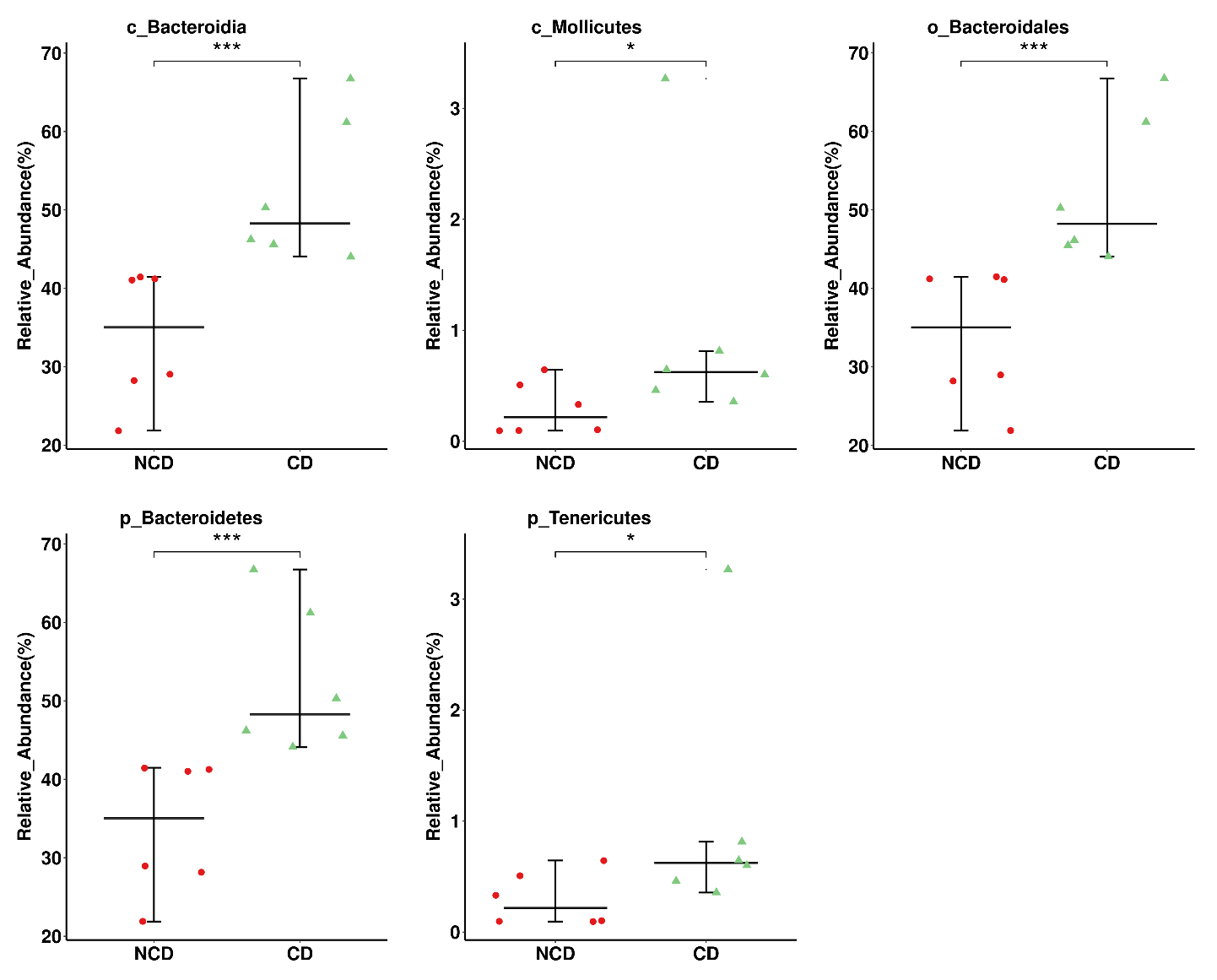


**Supplementary Fig. 6**

Relative abundance of gut microbiota. Data are presented as the mean ± SEM. *p < 0.05; **p < 0.01; ns, not significant.


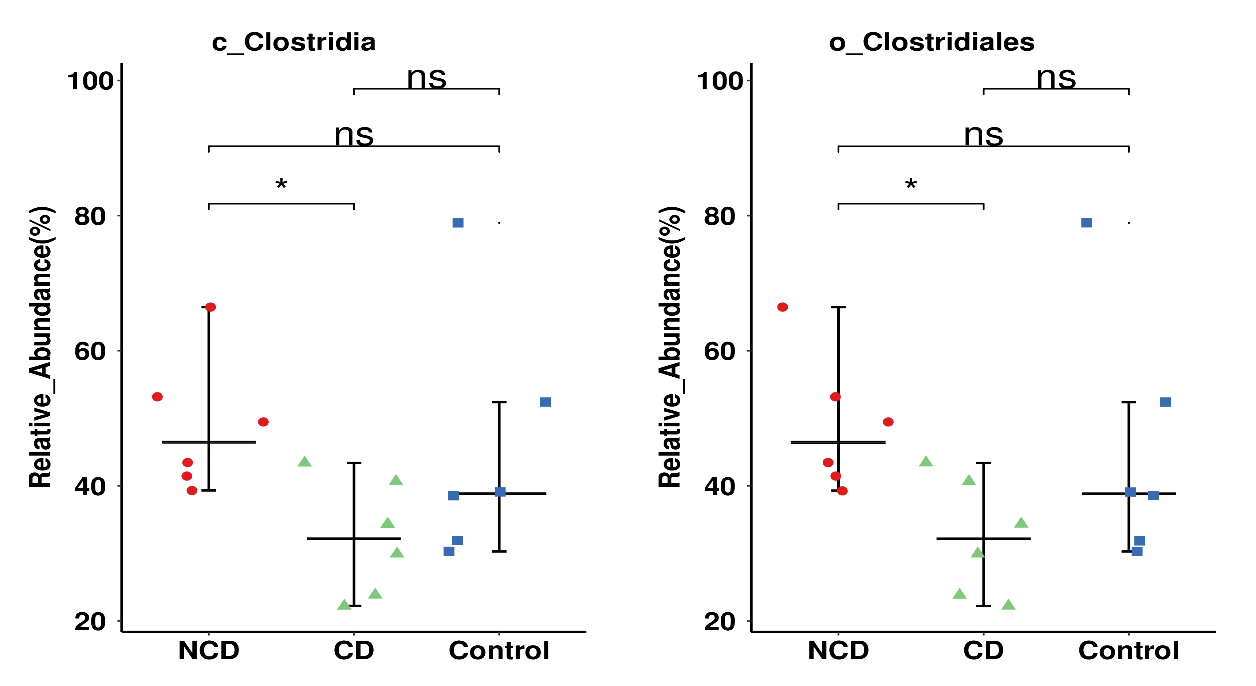


**Supplementary Table 1**

Df: degrees of freedom; SumsOfSqs: Total variance, also known as the sum of squared deviations; MeanSqs:SumsOfSqs/Df; F.Model: F.Model; R2: indicates the degree of explanation of sample variance by different subgroups, i.e. the ratio of the subgroup variance to the total variance, with a larger R2 indicating a higher degree of explanation of variance by subgroup; Pr(>F): indicates a p-value, less than 0.05 indicates a high degree of feasibility for this test, indicating a statistically significant difference at this grouping level.

| Df | SumsOfSqs | MeanSqs | F.Model | R2 | Pr(>F) |  |
| --- | --- | --- | --- | --- | --- | --- |
| Group_factor | 2 | 0.96 | 0.48 | 1.94 | 0.21 | 0.002 |
| Residuals | 15 | 3.69 | 0.25 | 0.79 |  |  |
| Total | 17 | 4.64 | 1.00 |  |  |  |
